# Interpretation for Individual Brain Age Prediction Based on Gray Matter Volume

**DOI:** 10.3390/brainsci12111517

**Published:** 2022-11-09

**Authors:** Jiancheng Sun, Zongqing Tu, Deqi Meng, Yizhou Gong, Mengmeng Zhang, Jinsong Xu

**Affiliations:** School of Software and Internet of Things Engineering, Jiangxi University of Finance and Economics, Nanchang 330013, China

**Keywords:** brain age prediction, complex network, gray matter volume, interpretable machine learning models

## Abstract

The relationship between age and the central nervous system (CNS) in humans has been a classical issue that has aroused extensive attention. Especially for individuals, it is of far greater importance to clarify the mechanisms between CNS and age. The primary goal of existing methods is to use MR images to derive high-accuracy predictions for age or degenerative diseases. However, the associated mechanisms between the images and the age have rarely been investigated. In this paper, we address the correlation between gray matter volume (GMV) and age, both in terms of gray matter themselves and their interaction network, using interpretable machine learning models for individuals. Our goal is not only to predict age accurately but more importantly, to explore the relationship between GMV and age. In addition to targeting each individual, we also investigate the dynamic properties of gray matter and their interaction network with individual age. The results show that the mean absolute error (MAE) of age prediction is 7.95 years. More notably, specific locations of gray matter and their interactions play different roles in age, and these roles change dynamically with age. The proposed method is a data-driven approach, which provides a new way to study aging mechanisms and even to diagnose degenerative brain diseases.

## 1. Introduction

It is well known that the human brain gradually changes with age; thus, there is a connection between the human brain and chronological age. For example, as we age, white matter volume (WMV) grows and then decreases, gray matter volume (GMV) decreases, and cerebrospinal fluid (CSF) gradually rises [1,2]. Magnetic resonance imaging (MRI), a non-invasive technique, makes it possible to use structural and functional neuroimaging data to predict age [3,4,5,6]. These studies can contribute to our understanding of the relationship between brain morphometry and age variation.

Aging causes significant changes in the structure and function of the brain, so the use of neuroimaging data to predict age has received considerable attention. Numerous studies are devoted to improving the accuracy of brain age prediction [3,7,8,9,10,11,12,13,14]. These studies have reported that the mean absolute error (MAE) of prediction for adults is less than five years. In previous studies, machine learning methods have been used with great success. For example, Varikuti et al. used non-negative matrix factorization (NNMF) for age prediction based on GMV to locate the relationship between brain regions and the predictions. Besides, their approach is sensitive to variations in typical structures in pathological aging [13]. Using support vector regression (SVR), Doosenbach et al. analyzed 238 (ages 7–30) scans of functional connectivity magnetic resonance imaging (fcMRI). Their findings show that long-range connections in the brain tend to grow with age, while short-range connections tend to decline. More recently, with the rapid development of deep learning, researchers have been applying it to age prediction [10,11,12,14]. Compared to traditional machine learning algorithms, deep learning models are capable of automatically extracting complex and abstract features, and as a result, typically achieve better performance. For example, Cole et al. found that both deep learning and Gaussian process regression (GPR) methods were able to achieve age prediction accurately for a given pre-processed data [14]. However, for a given raw MRI data, the deep learning approach showed outstanding advantages with an MAE of 4.65 years, while the GPR, on the other hand, can only achieve an MAE of 12 years.

In addition to using neuroimaging data to predict brain age, numerous studies have also considered how specific diseases are associated with brain age, which can help reduce the harmful effects of brain disease in later life [7,15,16,17,18,19,20]. Usually, many diseases are thought to exacerbate aging, and the brain is sensitive to deteriorating physical health. For example, people with mild cognitive impairment have older brains than their actual chronological age and have an increased risk of developing Alzheimer’s disease (AD) within three years [21]. Wang et al. developed a deep learning model in a study that included 5496 participants. They found that age differences were significantly associated with the risk of dementia. In addition, the difference between predicted age and true age may be a biomarker for screening for early dementia risk [16]. Recently, Jo et al. compared various algorithms of deep learning for AD diagnosis and found that the strategy of auto-encoder combined with a support vector machine (SVM) achieved the highest classification accuracy of 98.8% [22]. Although deep learning methods can usually improve diagnostic accuracy, their “black box” nature makes it difficult to interpret the results, which is one of the criticisms of deep learning. Boston University School of Medicine recently explored deep learning in the diagnosis and interpretability of AD, and its diagnostic accuracy outperformed that of a multi-institutional team of practicing neurologists [23]. Using the proposed probability map, they localized the brain regions involved in AD. However, since the probability map was obtained after dimension reduction using convolutional networks, the resolution was substantially lower than that of the original MR images.

The studies mentioned above suggest that changes in brain structure and cognitive function occur with age and that the changes are associated with functional decline and degenerative diseases. However, after clarifying these concerns, some other key issues have not been well addressed. For example, at the individual level, what is the relationship between age and the physiological structure of the brain? Is there some correspondence between age and voxel interactions? How do these effects evolve as we age? These questions also happen to be the motivation for the work of this paper. When faced with a problem, the primary goal is, of course, to solve it, but at the same time, you want to know how the task is being accomplished. For example, when a new medicine is introduced, people not only focus on the efficacy of the medicine itself, but also want to know what factors influence the efficacy, such as the patient’s age, gender, and the dose of the medicine. In a word, in the study of brain age prediction using neuroimaging data, we not only want to achieve age prediction but also to interpret how age changes.

Therefore, in the previous studies, most of the related works focused on achieving age or disease prediction in itself. These strategies usually take the voxel-wise information of the brain as a whole, which is a “black box” and lacks interpretability. Even when linear models were used to achieve average interpretability, the ability to interpret the individual is limited. In addition, fewer studies have been conducted on the dynamic analysis of the factors that influence aging. In our previous work, the construction of a snapshot network for the stock market was implemented using an interpretable model, while the prediction results were interpreted [24]. In this paper, new solutions to the above problems using GMV are proposed. In summary, at the scale of brain voxels, our study provides a novel approach to explain the relationship between gray matter and age based on interpretable machine learning models and complex network theory.

The rest of the paper is organized as follows: Section 2 describes the material and methods, including feature selection and interpretable machine learning models, and also presents voxel-based and network-based analysis methods; Section 3 shows the age prediction results and analyzes the relationship between gray matter and age; Section 4 provides a detailed discussion of the results, and Section 5 gives conclusions.

## 2. Materials and Methods

**Figure 1:**
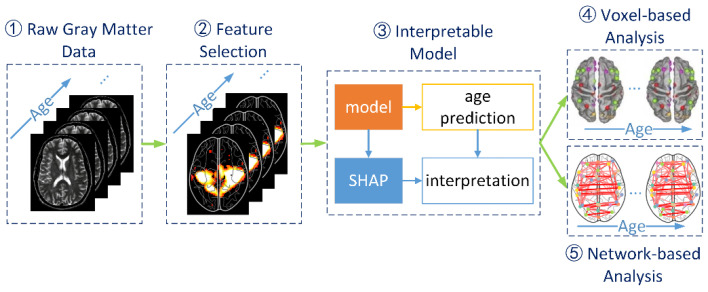
Workflow of the proposed method. The entire process is divided into five parts, starting with raw data preparation and feature selection, which is a data pre-processing stage. Then there is the interpretable machine learning model, which is tasked with achieving age prediction while providing interpretable parameters for subsequent analysis. Finally, for individuals, voxel-based and network-based analyses are implemented, as well as their dynamic evolutionary characteristics.

The workflow of the proposed method includes raw gray matter data preparation, feature selection, interpretable machine learning models, voxel-based analysis, and network-based analysis, as shown in Figure 1.

### 2.1. Data Sets

**Figure 2:**
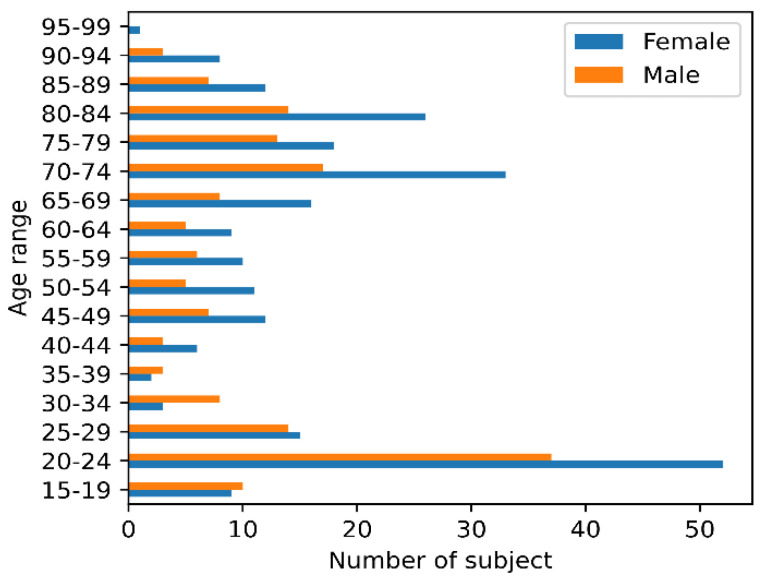
Age distribution for data set.

In this study, we use the classic version of the Open Access Series of Imaging Studies (OASIS): OASIS-1 dataset [25]. The dataset consists of a cross-sectional collection of 403 subjects (Thirteen outliers were removed from 416 subjects) aged 18 to 96. For each subject, 3 or 4 individual T1-weighted MRI scans obtained in a single scan were included. The subjects were all right-handed, both men and women. One hundred subjects aged 60 years or older had been clinically diagnosed with very mild to moderate AD. The age distribution of the subjects is shown in Figure 2, where more female subjects than male ones (160 males and 243 females). 

### 2.2. Data Preparation and Feature Selection

The data came from the OASIS-1 project. It has been pre-processed through a standard voxel-based morphometry (VBM) pipeline (using SPM8 and NewSegment), and we downloaded a DARTEL version of this data via Nilearn [26]. We first apply full width at half maximum (FWHM) filtering to the data and then perform feature selection. Note that in the following descriptions, both “voxel” and “feature” refer to the GMV unless otherwise specified. The reason is that they can be used to describe the problem more clearly in different cases. Additionally, the voxel implicitly has information about spatial location, so it is used to represent the GMV of the corresponding location.

Due to the high-dimensional nature of the raw data (the dimension is 902629), feature selection consists of two steps that aim to preserve the more informative features (voxels). A first step is an approach based on feature variance, which can be viewed as a rough feature selection. It does this by setting a certain threshold to remove features with too low between-subject variance. This approach considers only the features themselves without addressing the corresponding output, therefore it is an unsupervised learning approach to feature selection. In the second step, features based on the idea of univariate statistical tests are selected. The statistical-based approach first uses statistical indicators to assess the relationship between each input feature and the target variable and then selects the input features that have a strong relationship with the target variable. Here we use the classical univariate feature selection method based on the F-test, which estimates the degree of linear dependence between two random variables.

### 2.3. Interpretable Machine Learning Model

Using the selected features, an interpretable model can be built and trained. The primary strategy is to implement age prediction based on eXtreme Gradient Boosting (XGBoost) [27] and combine it with SHapley Additive exPlanations (SHAP) [28] to provide interpretability.

#### 2.3.1. Formulation of Age Prediction 

Based on the selected features, we make age predictions and related interpretations. Note that age is a continuous variable; hence, we use a regression model rather than a classification one. Here the regression model considers y (age) as a function of x (GMV) and β (model parameters):

(1) y=f(x,β)+e

where e denotes model additive random noise. Our goal is to estimate the function f(⋅) so that it most closely fits the training data:

(2) $$T=\left\{\left(x_{1},y_{1}\right),\left(x_{2},y_{2}\right),\cdots ,\left(x_{N},y_{N}\right)\right\}$$

where N is the number of subjects, $x_{t}\in X={\mathbb{R}}^{M}$ is the *t*-th feature vector, $y_{t}\in Y=\mathbb{R}$ is the observed age corresponding to $x_{t}$, and the data pair $\left(x_{t},y_{t}\right)$ is called a sample training point. This is a typical supervised regression problem, and here XGBoost is used to solve the regression. Using XGBoost is partly because of its strong nonlinear fitting ability and partly because of its interpretability, which is a basis for constructing the network later using the SHAP.

#### 2.3.2. SHAP Model for Interpretation

SHAP is derived from game theory and belongs to the after-the-fact approach to explaining complex machine-learning models [29]. Here we use SHAP to interpret the XGBoost model and its predictions. For individuals, the explanation for age is developed from two aspects: the feature itself and the feature interactions.

For each training sample, the model generates a predictive value, the SHAP value being the value assigned to each feature in that sample. Assuming that the *t*-th sample is $x_{t}$, the i-th feature of the $x_{t}$ is $x_{t,i}$, the model’s predictive value for that sample is $y_{t}$, and the baseline of the entire model (usually the mean of the target variables for all samples) is $\phi_{0}$, then the SHAP value obeys the following additive explanatory model:

(3) $$y_{t}=\phi_{0}+\overset{M}{\underset{i=1}{\sum }}\phi_{i}\left(x_{t,i}\right)$$

where M is the number of features and $\phi_{i}\left(x_{t,i}\right)$ is the SHAP value of $x_{t,i}$. Intuitively, $\phi_{1}\left(x_{t,1}\right)$ is the contribution of the 1st feature in the *t*-th sample to the final predicted value of $y_{t}$, and when $\phi_{1}\left(x_{t,1}\right)>0$, it means that the feature raises the predicted value and therefore has a positive effect; conversely, it indicates that the feature makes the predicted value lower and has the opposite effect. The SHAP value $\phi_{i}\left(x_{t,i}\right)$ is defined as [28]:

(4) $$\phi_{i}\left(x_{t,i}\right)=\underset{S\subseteq \left\{x_{t,1},\ldots ,x_{t,M}\right\}\backslash \left\{x_{t,i}\right\}}{\sum }\frac{\left|S\right|!\left(M-\left|S\right|-1\right)!}{M!}\cdot \left(f\left(S\cup^{\;}\left\{x_{t,i}\right\}\right)-f\left(S\right)\right)$$

where $\left\{x_{t,1},\ldots ,x_{t,M}\right\}$ is the set of all the input features, $\left\{x_{t,1},\ldots ,x_{t,M}\right\}\backslash \left\{x_{t,i}\right\}$ is the set of all input features excluding $\left\{x_{t,i}\right\}$, and f(S) is the prediction of feature subset S. Traditional feature importance can only tell us globally which feature is important, but we do not know how that feature of an individual sample affects the prediction of the results. The most striking advantage of the SHAP value is that it not only reflects the influence of the features in each sample but it is also able to show the positive and negative impact. Briefly, we can now use $\phi_{i}\left(x_{t,i}\right)$ to estimate the effect of each feature on an individual’s age.

In addition to the features themselves, we are interested in the effect of the interaction values between features on the output $y_{t}$. The interaction $\phi_{i,j}\left(,\right)$ between feature $x_{t,i}$ and $x_{t,j}$ is defined as:

(5) $$\phi_{i,j}\left(x_{t,i},x_{t,j}\right)=\underset{S\subseteq \left\{x_{t,1},\ldots ,x_{t,M}\right\}\backslash \left\{x_{t,i},x_{t,j}\right\}}{\sum }\frac{\left|S\right|!\left(M-\left|S\right|-2\right)!}{2\left(M-1\right)!}\delta_{i,j}\left(S\right)$$

when i≠j, and

(6) $$\delta_{i,j}\left(S\right)=f\left(S\cup^{\;}\left\{x_{t,i},x_{t,j}\right\}\right)-f\left(S\cup^{\;}\left\{x_{t,i}\right\}\right)-f\left(S\cup^{\;}\left\{x_{t,j}\right\}\right)+f\left(S\right)\;\;\;$$

when i=j, we have

(7) $$\phi_{i,i}\left(x_{t,i},x_{t,i}\right)=\phi_{i}\left(x_{t,i}\right)-\underset{i\neq j}{\sum }\phi_{i,j}\left(x_{t,i},x_{t,j}\right)\;\;\;\;$$

Therefore, the output of the model is the sum of $\phi_{i,j}\left(,\right)$:

(8) $$y_{t}=\overset{M}{\underset{i=1}{\sum }}\overset{M}{\underset{j=1}{\sum }}\phi_{i,j}\left(x_{t,i},x_{t,j}\right)\;\;\;\;\;$$

Equations (3) and (8) describe the mechanisms by which features themselves and feature interactions affect model output, respectively. In practice, we can directly use (4) and (5) to calculate the SHAP values and SHAP interactions, respectively.

### 2.4. Voxel-Based and Network-Based Analyses

Based on the SHAP values and SHAP interactions, voxel-based and network-based analyses can be performed, respectively.

#### 2.4.1. Voxel-Based Analyses 

For individuals, voxel-based analyses using SHAP values are straightforward. Referring to (3), intuitively, $\phi_{i}\left(x_{t,i}\right)$ is the contribution of the *i*-th voxel to the output $y_{t}$ (age) for the *t*-th subject. Thus, we use $\phi_{i}\left(x_{t,i}\right)$ to analyze the relationship between voxels and age. As mentioned earlier, the significant advantage of the SHAP value is that it reflects the influence of the features in each sample on the output, and it also shows the positive and negative nature of the influence. However, it should be clear that $\phi_{i}\left(x_{t,i}\right)$ is not just a role of $x_{t,i}$ itself, but also includes interactions between $x_{t,i}$ and other voxels. To understand this more clearly, we rewrite (7) as:

(9) $$\phi_{i}\left(x_{t,i}\right)=\phi_{i,i}\left(x_{t,i},x_{t,i}\right)+\underset{i\neq j}{\sum }\phi_{i,j}\left(x_{t,i},x_{t,j}\right)\;\;\;\;$$

where $\phi_{i,i}\left(,\right)$ is the SHAP value for $x_{t,i}$ itself and $\phi_{i,j}\left(,\right)$ is the interactions between $x_{t,i}$ and other features. From (9), we can see that $\phi_{i}\left(x_{t,i}\right)$ is the composite of the function of $x_{t,i}$ and its interactions with other features. Combining (3) and (9), it can be seen that the sum of all $\phi_{i,i}\left(,\right)$ does not equal $y_{t}$, which is why $\phi_{i}\left(x_{t,i}\right)$ is used instead of $\phi_{i,i}\left(,\right)$ for voxel-based analysis.

In addition to targeting individuals, we can also study the dynamic relationship between SHAP values and age change. Using dimensionality reduction techniques, the SHAP value of each individual is reduced to a lower dimensional space for visualization and qualitative analysis. Here, we use classical multidimensional scaling (MDS) for dimensionality reduction [30].

#### 2.4.2. Network-Based Analyses

The motivation for the network-based analysis is to explore whether the correlation between voxels is also related to aging. Similar to the voxel-based analysis, we first perform a network analysis for individuals and then analyze the dynamic properties of the network structure.

A network can usually be represented as a graph (here we only focus on the simple undirected graph), which is a data pair G=(V,E), where V is a set of vertices and $E\subseteq \left\{\left\{p,q\right\}|\left(p,q\right)\in V^{2}\wedge p\neq q\right\}$ is a set of edges. In this paper, V is the set of voxels, while E consists of SHAP interactions. Specifically, regarding (5), the interactions form an M×M weighted adjacency matrix $A=\left(a_{i,j}\right)$ which is the mathematical expression for E, where $a_{i,j}=\phi_{i,j}\left(x_{t,i},x_{t,j}\right)$. Thus far, in combination with (8), we can use A to analyze the effect of voxel interactions on individual age, and additionally visualize A for qualitative analysis.

For N subjects, let $A=\left\{A_{1},A_{2},\ldots ,A_{N}\right\}$, and then we can analyze the dynamic properties of the individual networks using A. The premise of performing a dynamic analysis is to measure the similarity between individual networks. Thus, first, we transform $A_{t}$ by regularizing graph Laplacian into a symmetric positive definite (SPD) matrix ${\bar{A}}_{t}$ [31]

(10) $${\bar{A}}_{t}=\left(D_{t}-A_{t}\right)+\lambda I\;\;$$

where λ>0 is a regularization parameter, I is the identity matrix and $D_{t}$ is the degree matrix. With $\left|A_{t}\right|=n$ and $v_{i}\in V$, $D_{t}=diag\left(d\left(v_{1}\right),d\left(v_{2}\right),\cdots ,d\left(v_{n}\right)\right)$ is a n×n diagonal matrix and the degree $d\left(v_{i}\right)$ of $v_{i}$ is defined as $d\left(v_{i}\right)=\sum_{j=1}^{n}w_{ij}$, where $w_{ij}$ is the weight between $v_{i}$ and $v_{j}$. Then the geodesic distance between two SPD matrices can be defined as [32]:

(11) $$d\left({\bar{A}}_{i},{\bar{A}}_{j}\right)=\mathrm{tr}\left(\log^{2}\left({\bar{A}}_{i}{}^{-\frac{1}{2}}{\bar{A}}_{j}{\bar{A}}_{i}{}^{-\frac{1}{2}}\right)\right)$$

where tr is the trace operator for the matrix. Using (11), the similarity between two networks can be measured, and thus a new network can be constructed, i.e., the adjacency matrix $B=\left(b_{i,j}\right)$, where $b_{i,j}=d\left({\bar{A}}_{i},{\bar{A}}_{j}\right)$. In other words, B represents a network of networks in which each node represents an individual network. Based on the network B, we can study the dynamics of individual networks and the relationships between them at a macro level.

## 3. Experiments and Results

In this section, age prediction is first implemented using XGBoost, and then the prediction is interpreted using the SHAP model. The relationship between gray matter and age is interpreted in terms of voxels and networks, respectively, which include individual as well as dynamic interpretation.

### 3.1. Age Prediction

As can be seen in Figure 2, there are more females than males in the data set. To investigate the effect of sex on age prediction, here we first give a statistical analysis between age, sex, and GMV, followed by age prediction for all subjects, males and females, separately.

#### 3.1.1. Relationship between Aging, Sex, and GMV

**Figure 3:**
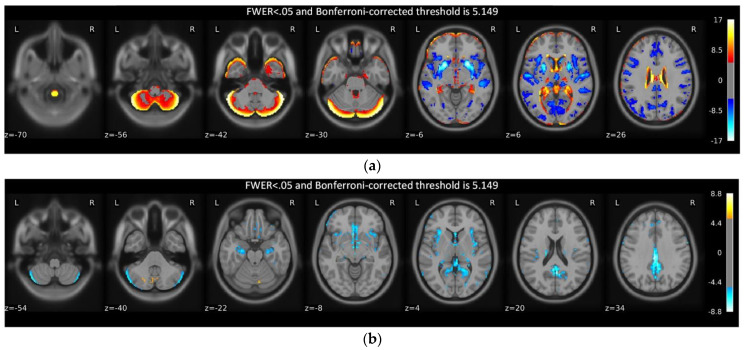
Statistical analysis results of GLM. The images are Bonferroni−thresholded z−statistic maps which show the statistical significance of effect size (weights of the linear model). The Bonferroni procedure was used to correct significance to reduce false positives, and the corrected threshold is 5.149 under FWER <0.05 (Family−Wise Error Rate). (**a**) Age effect on GMV; (**b**) Sex effect on GMV.

In our work, GMV was used to predict age. To discern whether sex has a significant effect on age prediction, it is necessary to investigate the relationship between aging, sex, and GMV. The generalized Linear Model (GLM) has been used in most areas of neuroimaging to build models and perform statistical hypothesis testing. The GLM is fundamentally a linear model, which is possible to analyze the degree of effect of the explanatory variables (age and sex) on the observed signal (GMV). Therefore, we performed a standard second-level GLM analysis to investigate the relationship between aging, sex, and GMV. The design matrix consisted of age, sex, and intercept, and then the effect of aging and sex on GMV can be studied by estimating the weights (effect sizes) of the linear model. Based on the modeling of the dataset including all subjects, Figure 3 shows the effects of age and sex on GMV.

As seen in Figure 3a, age has a significant effect on GMV; but as seen in Figure 3b, sex has few effects on GMV, a result that is consistent with previous studies. For example, Carla et al. found that “When TIV effects are ruled out using appropriate adjustment methods, few sex differences (if any) remain statistically significant” [33]. Since GMV is used to predict age, the weak relationship between sex and GMV appears to indicate that sex has little effect on prediction. To further investigate this observation, In the next section, age prediction will be performed using all subjects, males, and females, separately.

#### 3.1.2. Age Prediction Using XGBoost Model

As described in Section 2.1, we first performed FWHM (FWHM = 6) smoothing filtering on the data, then performed the first feature selection based on variance (threshold = 0.01), and finally pass the second feature selection based on F-test. After these steps, the number of feature dimensions was reduced from 902629 to 2000. To illustrate the difference in age prediction by sex, in addition to using all subjects, we additionally performed separate predictions for males and females. In each prediction, the feature-selected dataset was randomly divided into training and test sections, with 15% of the test sample. XGBoost was used for age prediction, and additionally, linear SVM was used for performance comparison.

**Figure 4:**
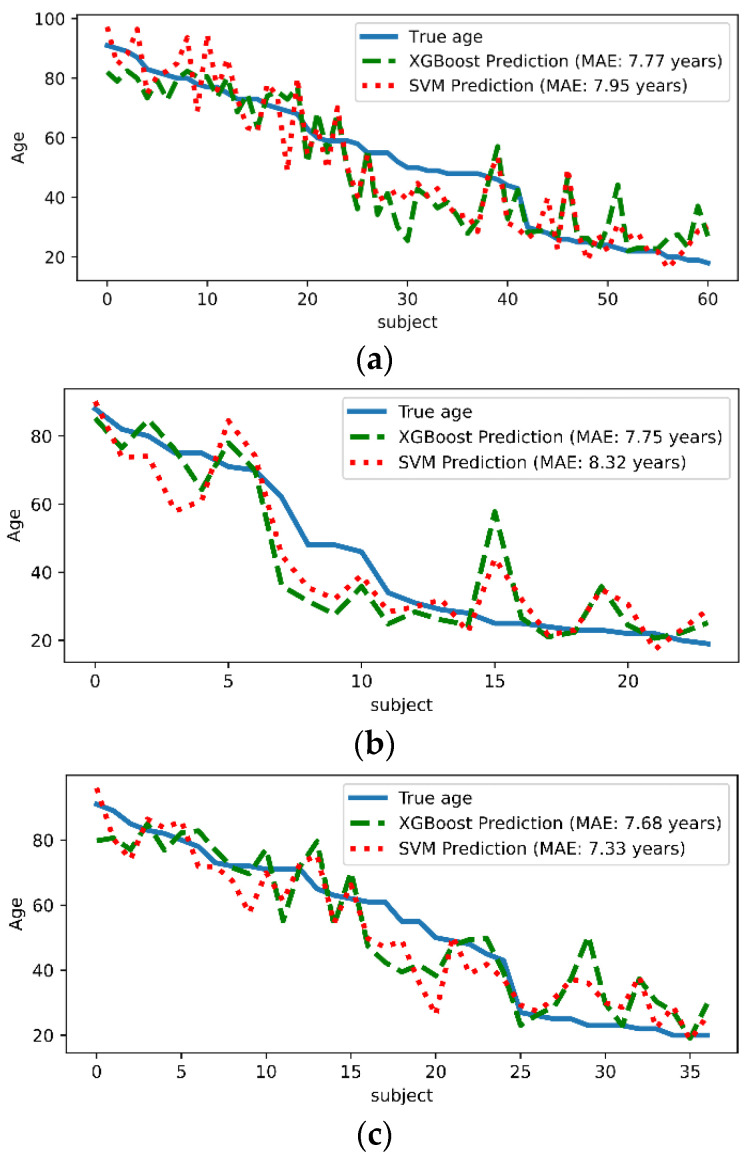
Age prediction of XGBoost and linear SVM. (**a**) Prediction results for all subjects, (**b**) prediction results for males, and (**c**) prediction results for females.

The prediction results are shown in Figure 4, where one can see that the MAE for XGBoost in three cases is 7.77, 7.75, and 7.68 years, respectively. Similarly, the predictions of linear SVM for different subject groups are close (7.95, 8.32, and 7.33). These results suggest that sex makes very little difference in age prediction. Note that although the predictive performance of the linear SVM is comparable, it cannot explain the interactions between features compared to XGBoost. For comparison with classical methods, the prediction results of the representative methods are presented in Table 1. It is seen that the accuracy of the CNN-based deep learning methods is higher at 4.16 and 5.55 years, respectively. The accuracy of our method is comparable to that of GPR and RVR. With the advantage of big data and automatic feature extraction, deep learning is generally state-of-the-art. Although there is still a gap between the predictive performance of XGBoost and the state-of-the-art, it should be noted that the primary goal of this paper is the explanatory power of the model, rather than just improving the predictive performance. 

### 3.2. Voxel-Based Results

For individuals, the goal of voxel-based analyses is to explore and explain the relationship between voxels themselves and aging. We will address both the individual itself and the dynamic characteristics of the individual.

#### 3.2.1. Individual Voxel-Based Analyses

**Figure 5:**
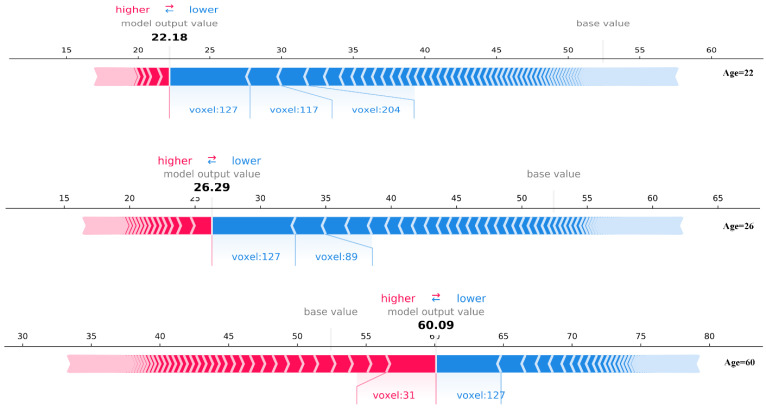
Force-plot of three subjects. The arrows represent the SHAP values corresponding to the voxels. Red and blue mark the positive and negative SHAP values, representing the positive and negative contributions of each voxel to the model output, respectively, while the length of the arrows represents the degree of the contribution. The horizontal coordinates are labeled age, the model output is the age prediction, and the number to the right of each arrow bar is the true age.

After training on age prediction and the SHAP model, the SHAP values in (4) were used for our analysis. XGBoost is known to be a tree-based boosting algorithm so that it can evaluate the importance of features. Note that for correlated features, XGBoost selects the most important features and ignores redundant ones. Usually, neighboring voxels have a strong correlation, so a selected voxel can be regarded as the peak voxel in a region. Here, by controlling the depth of the decision tree and ranking the voxels according to their importance, the top 310 voxels were finally selected as peak ones. Three subjects were selected according to the progression of age and plotted the SHAP force plot shown in Figure 5, precisely the illustration of (3) [34]. In the figure, the SHAP value can be visualized as a “force”, i.e., the SHAP value corresponding to each feature is a force that increases or decreases the prediction. The force plot shows how each feature pushes the model output from the base value (the average of all predictions) to the final output of the model. Here, each SHAP value is plotted by an arrow, with red (positive values) indicating features that push up the prediction and blue (negative values) indicating features that pull down the prediction. Additionally, the arrow’s length represents the strength of pushing up or pulling down the prediction.

For descriptive convenience, the voxels were numbered in order. In Figure 5, we start with the first subject (age 22) as an example, where voxels 127, 117, and 204 are the three most essential voxels in terms of their effect on age. Among them, voxel 127 makes the subject approximately five years younger than the baseline value (length of the arrow), while the other voxels also contribute differently to the subject’s current age. Overall, the power of pulling down age (sum of lengths of blue arrows) is much greater than that of pushing up age (sum of lengths of red arrows). Let us go back to subject 3 (age 60) in Figure 5, and overall, the power to push up age is greater than the power to pull down age. Simultaneously, voxel 127 remains the most important force for pulling down age, while voxel 31 is the leading force for pushing up age. Looking at the three subjects, it can be seen that with increasing age, the power of the age-pushing voxels increases, compared to the power of the age-pulling voxels decreases. This phenomenon is consistent with the rule of aging. In summary, the SHAP values corresponding to the features give us a quantitative way to interpret the age of individuals from the voxel scale.

**Figure 6:**
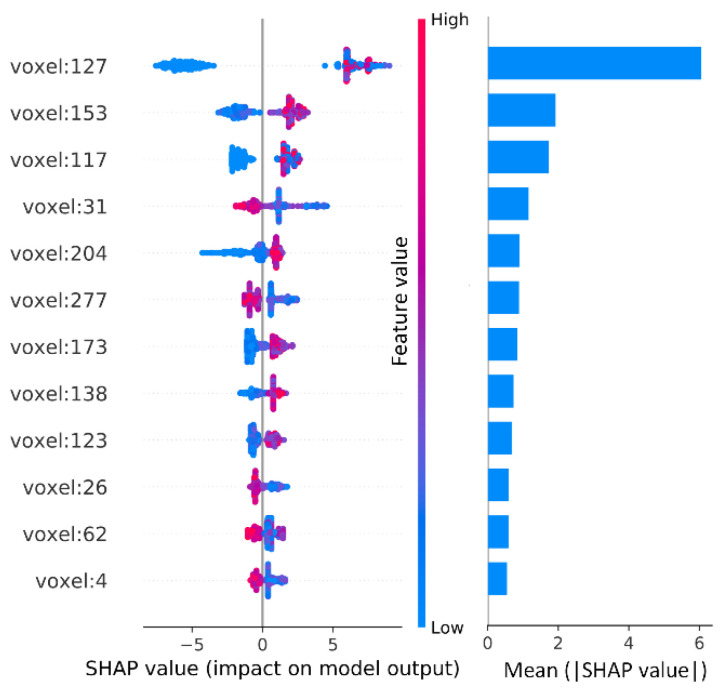
Summary plots (**left**) and feature importance (**right**) of 12 important voxels. The summary plots are a set of beeswarm plots where each row represents a feature and each point corresponds to a subject. The position of the points on the x-axis shows the effect of the feature on the age prediction of the subject. When multiple points fall at the same x position, they stack up vertically to show density. Here the feature values indicate the amount of GMV. The bar chart on the right represents the importance of each feature, and each bar is the average of all SHAP absolute values in the corresponding row.

Using SHAP values, the features globally can be analyzed and visualized, in addition to individual features. SHAP summary plots and feature importance are shown in Figure 6. The feature importance is a global attribute of the feature and does not contain detailed information about the individual. To get more detailed information, we need to check the summary plots. The summary plots give SHAP values for each feature in each individual, which can be used to better understand the overall pattern and discover outliers.

From Figure 6, it is clear that voxel 127 is the most important feature, and its role in individuals is different, pulling down age in some subjects and the opposite in others. For voxel 127 it also found that high feature values usually push up age, but this is not a universal rule. For example, in the case of voxels 31, 277, 26, 62, and 4, where high feature values are negatively correlated with age. Besides, it notes that the distribution of some feature values is long-tailed, e.g., voxels 31 and 204. Although the global importance of these voxels is not very high, the long tails imply that they are extremely important for specific individuals.

**Figure 7:**
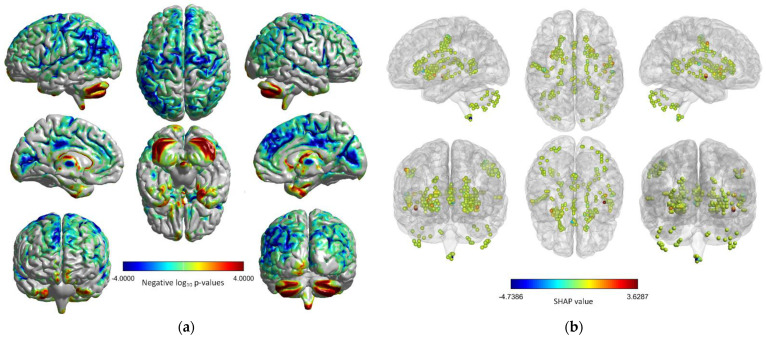
Volume−to−surface mapping and voxel SHAP values for the subject aged 63: (**a**) Brain mapping with mass univariate. Massively univariate group analysis with permuted OLS is performed to fit the test variables (age) independently to the target variable (brain imaging signals). Permutation tests are used to assess the significance of the relationship between the test variables and the target variables [41], and a max−type procedure is used to obtain family−corrected *p*-values; negative $\log_{10}$ *p*-values are displayed as heat maps on the cortical surface, and (**b**) voxels with high SHAP values, which are identified by tiny nodes. The graphs were visualized with BrainNet Viewer [42].

While Figure 5 and Figure 6 illustrate to some extent which voxels have an effect on age, they are not yet intuitive enough. Therefore, Figure 7b shows the physical locations of the voxels with high SHAP values to better demonstrate and analyze the role of these voxels. Since these voxels are peak voxels, they are distributed in a discrete manner. In addition, for comparison, we used massively univariate group analysis with permuted Ordinary Least Squares (OLS) criterion for all voxels and projected significance onto the cortex in Figure 7a, which allows us to see essential regions more clearly. In fact, the relationship between GMV and age in different brain regions has been well-studied [35,36,37]. However, the resulting age-sensitive brain regions are not entirely consistent due to the methods used and the different data sets. As shown in Figure 7a, it can be found that the significant regions mainly include the temporolimbic region, cingulate gyrus, insula, cerebellum, brain stem, and lateral temporal cortex, which is generally consistent with the results of Terribilli et al. [38].

In Figure 7b, we consider the nodes as peak voxels, i.e., they have high SHAP values compared to other voxels. The nodes are roughly symmetrically distributed and are consistent with the statistical map in Figure 7a. It is important to note here that the SHAP values in Figure 7b are individual-based, whereas the univariate analysis in Figure 7a is a group statistical approach. Therefore, Figure 7b can be used to perform an individual-specific analysis and found out which voxels are correlated with the subject’s age. As seen in Figure 7b, the voxels are distributed in the following regions: Sylvian fissure, superior temporal sulcus, inferior frontal sulcus, precentral sulcus, central sulcus, cerebellum, and brain stem. In particular, the Sylvian fissure, inferior frontal sulcus, and brain stem include significant voxels. For example, the voxel (red node) in the superior temporal has a positive effect on the age of the subject. In addition, it can be found in both Figure 7a and Figure 7b, voxels located at the brainstem play an important role, and the most important voxel 127 mentioned earlier is also located here. In fact, studies have shown that the volume and myelin content of the brainstem increase [39] and decrease [40] with age, respectively.

#### 3.2.2. Dynamic Analysis and Visualization of SHAP Values

**Figure 8:**
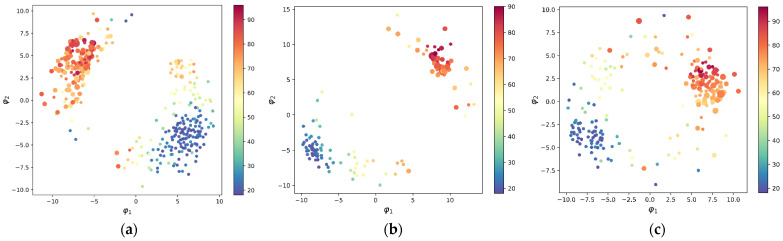
Two-Dimensional visualization of SHAP values for individuals of different ages. The color bar indicates the age, and the size of the dots from small to large indicates the subject’s four levels of dementia, which are no dementia, very mild dementia, mild dementia, and moderate dementia. (**a**) Visualization for all subjects, (**b**) visualization for males, and (**c**)visualization for females.

As we age, it is necessary to investigate the dynamic relationship between SHAP values and age. All SHAP values of an individual are in a high-dimensional space. Due to the invisibility of the high-dimensional space, the MDS in Section 2.4 was used to reduce the SHAP values of each subject to two-dimensional space for visualization. Based on the prediction models, the visualization results for all subjects, males, and females, are presented separately in Figure 8 to investigate the differences in the distribution patterns of the different groups.

In the figure, each dot corresponds to a subject. From Figure 8a–c, it is seen that the subjects are roughly divided into two clusters at baseline age 52.5, which correspond to the older and younger groups, respectively. This is because the predicted age of each subject is obtained by summing the baseline age with the other SHAP values (see (2)). In addition, it can be seen that subjects of similar age are usually close together as well, especially the groups under 30 and over 70 years of age clustered more closely together. Overall, except for very few outliers, SHAP values change smoothly with age, i.e., individuals of similar age usually have similar SHAP values. From the above observations, it can be seen that the pattern distribution is very similar in the three figures, except for some detailed differences. The results also indicate that the specificity of sex for age prediction is not obvious.

In addition to age, there has been concern about whether GMV is associated with degenerative brain disease. Although the main work in this paper is not focused on brain diseases, it can still find some illuminating phenomena in Figure 8. This is because age, GMV, and brain disease usually correlate with each other. For example, the figure shows that the vast majority of subjects with dementia are clustered closely together, but there are a few exceptions. This phenomenon suggests that there is something special about the GMV of these patients compared to other subjects.

### 3.3. Network-Based Results

Unlike the voxel-based analysis, the network-based approach explores the effect of voxel interactions on age. We first target individuals to analyze the effect of voxel interrelationships on age and then investigate how the voxel network changes with age.

#### 3.3.1. Individual Network-Based Analysis

In the previous section, the relationship between the voxels themselves and age has been analyzed. However, according to (8), it can also find that the interaction $\phi_{i,j}\left(,\right)$ between the voxels is also related to age. That is, in addition to the voxels themselves, the interactions between voxels give additional information about aging. Although these interactions are not physical connectivities, they can still provide us with important information so that these interactions can be considered functional ones, and this is somewhat similar to functional connectivity in fMRI [43].

**Figure 9:**
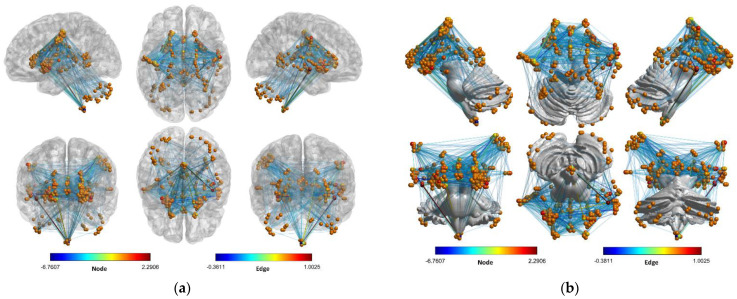
Voxel-based network for the subject aged 63. Edges indicate the effect of interactions between voxels on age. If the interaction between two nodes contributes significantly to age, there is an edge between the nodes. The colors of nodes and edges represent the strength of the contribution, respectively, where positive or negative values signify the direction of the contribution. (**a**) Network with a brain as reference, (**b**) network with cerebellum as reference. The graphs were visualized with BrainNet Viewer [42].

The contribution of the voxels themselves and the interactions between the voxels to age are shown in Figure 9. To identify the spatial location of voxels more easily, the voxel network with reference to the brain and cerebellum are shown in Figure 9a,b, respectively. It is important to clarify here that the role of the voxels here is different from that in Figure 7. To illustrate this point, we refer to (9), where $\phi_{i}()$ and $\phi_{i,i}\left(,\right)$ represent the contribution of the voxels themselves to age in Figure 7 and Figure 9, respectively. That is, $\phi_{i}()$ is the combined result of $\phi_{i,i}\left(,\right)$ and the interactions between the voxels.

Another important issue that needs to be addressed is clarifying the relationship between voxel interaction and age. As can be seen in Figure 9, not all interactions between voxels are related to age, and only the edges with significant contributions are shown. The network has a roughly symmetrical topology, with edges between voxels in the left and right hemispheres, in addition to edges between voxels within the hemispheres. Besides, some remarkable structures can be observed. For example, the edges between voxels near the brain stem and other voxels are radially distributed, suggesting that voxels around the brain stem play a pivotal role in their contribution to age. In addition, a few edges show significant color (tending to dark blue or dark red), while most edges show light blue, suggesting that the contribution of most edges to age tends to be consistent. Generally, if a voxel is important, the edge it connects also appears to be important. Based on Figure 9, we are able to analyze the relationship between voxels and age from a new perspective, i.e., in addition to the voxels themselves, the correlation between the voxels is also a very important factor.

#### 3.3.2. Dynamic Network Analysis with Age

**Figure 10:**
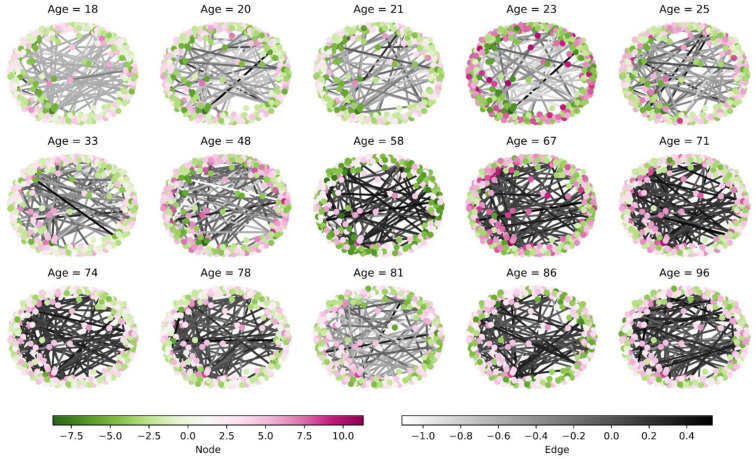
Dynamic network of SHAP interaction values. The individual networks are arranged in increasing order of age, with the top of the network identifying its age. The two color bars at the bottom of the figure identify the magnitude of the SHAP values of the nodes and edges respectively.

For each individual, a network of SHAP interaction values is available. Thus the dynamic properties of the network structure for the individual’s age can be investigated. We selected 15 subjects from the sample in order of age and showed the network of SHAP interaction values at different ages in Figure 10. Note that to highlight the topology of the network based on its connectivity, the location of the network nodes here is not the actual spatial location of the voxels. Besides, for comparison purposes, we fixed the positions of the nodes and used the SPRING layout in NetworkX [44].

In Figure 10, from the perspective of nodes, it can observe that nodes in the same position (voxels) play different roles for different subjects and ages. For example, the same nodes have opposite roles (pushing up or pulling down age) when they are young and when they are old, while the role of some nodes remains stable. The change of these nodes with age allows us to determine which voxels are sensitive to changes in age.

As can be seen from the figure, the role of the edge changes accordingly with age. For the individuals themselves, the role of the edges is also different. For example, for middle-aged subjects with 48, both push-up and pull-down age edges are present, and the number of edges in both categories does not differ significantly. Considering different ages, it can be seen that as the age increases, the number of edges pushing up the age increases in the vast majority of subjects (the color of the edges gradually deepens). Thus, we can draw some rules from the process of dynamic changes of edges. Using these rules, it can find some subject specificities. For example, the pattern of edges in the subject aged 81 is similar to that of young people and inconsistent with that of most elderly ones, which leads us to elucidate the reasons behind the phenomenon.

#### 3.3.3. Network of SHAP Interaction Networks

**Figure 11:**
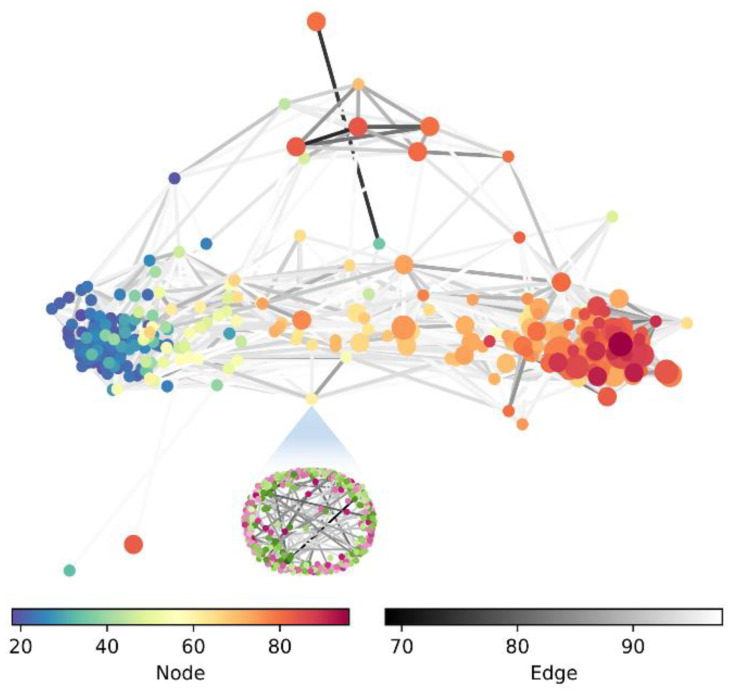
Network of SHAP interaction networks. Each node represents a SHAP interaction network of a subject (below the figure is an example of the network corresponding to a node), and the two color bars represent the age of the node and the connection strength of the edge (black represents high strength), respectively. The size of the dots from small to large indicates the subject’s four levels of dementia, which are no dementia, very mild dementia, mild dementia, and moderate dementia.

Based on the interaction networks, the effects of voxels themselves and interactions between voxels on age can be studied at a detailed level. However, too much detail can obscure the macroscopic information and make us “see the trees but not the forest”. Therefore, we measure the distance between two SHAP interaction networks according to (11), which constitutes a new network, i.e., a network of networks. That is, as shown in Figure 11, each node in the network also represents a network. This allows us to study the relationship between individuals at a macro level. To illustrate the role of edges, the effect of the SHAP values of the nodes is removed, which means that the diagonal values of the adjacency matrix $A_{t}$ are set to zeros. Moreover, 25% of the edges were preserved by setting the distance threshold to highlight the topology of the network.

In Figure 11, from an age perspective, it is clear that the younger and older subjects are divided into two tight clusters and begin to move toward the older group around age 40, which is largely consistent with our a priori common knowledge. Since the role of voxels is removed when constructing the network, this topology accounts for the effectiveness of edges in SHAP interaction networks to some extent. Using the network in the figure, some additional meaningful structures and phenomena can be discovered quickly. For example, it can find a small number of isolated subjects (two subjects located in the lower part of the figure and one subject in the upper part), which makes it interesting to explore why this specificity occurs. In addition, although the goal of the proposed model is not to predict dementia, it can observe a strong association between several subjects with dementia in the upper part of the figure, which suggests a correlation between age and dementia. There is also the possibility that it can study the similarity between a particular subject and its neighbors (nodes with direct connectivity) to find some meaningful patterns.

## 4. Discussion

In the current study, machine learning models have been used to achieve age prediction. More importantly, the relationship between age and brain structure was interpreted for individuals from the voxel scale.

The first step of our method is to achieve age prediction. We must admit that our method is not yet the best in age prediction compared to the deep learning models [10,11,12,14]. However, Due to the “black box” nature of deep learning models, it is generally difficult to interpret the results accordingly. In comparison, the highlight of the current study is the interpretation of the brain structure behind age, not just age prediction. After all, when we age, we care more about what happens to the brain structure, which is especially meaningful for each individual.

To obtain the interpretability of age-related changes, new methods are needed to better capture changes in brain structure and cognition. In previous studies, linear additive machine learning models have been widely used in the analysis of neuroimaging data [45,46,47,48,49,50], and one of the main reasons is that the additive weights of linear models are interpretable. However, in the context of brain aging, the “weight maps” obtained from linear machine learning models are complex and do not provide a straightforward explanation [45]. The additive weights, while giving the importance of different features (parts of the brain) for prediction in an average sense, do not give a more specific interpretation for individuals. Additionally, studies have shown that age-related brain changes are nonlinear, spatially distributed, and vary between individuals [51,52,53]. Thus, new methods are needed to better analyze and interpret individual brain age and associated brain diseases.

In the current work, the key points mentioned above are addressed, and then the relationship between age and brain structure is explained from an individual perspective and at the voxel scale. Specifically, our contributions include the following three aspects:

- For individuals, we give quantitative interpretations of the relationship between aging and GMV. For example, it can find a positive contribution of GMV of some specific locations to age.
- In addition to the GMVs themselves, we consider the interactions between GMVs that construct a complex network for each individual. The effects of these networks on age-related changes are then investigated.
- Based on dimensional reduction and network similarity, we investigate the dynamic properties of GMV as well as brain network changes with age.

Dementia was addressed in the current findings, mainly because dementia and age are usually closely related. However, this paper focused mainly on the prediction of age, meaning that dementia was not included as a predictor. Some previous studies have focused on the prediction of degenerative diseases [22,23]. Similar to age prediction, the proposed method can be directly applied to the prediction of degenerative diseases if the disease is the predictive target, and additionally the relationship between disease and brain structure can be interpreted. In addition to degenerative diseases, our work has potential applications to psychiatric disorders, such as depression. Liston at Cornell University acquired fMRI images of over a thousand subjects, 40% of whom were depressed [54]. Considering the heterogeneity of depression itself, finding biomarkers or other indicators of depression as new diagnostic criteria is a challenging task. We can try to find biomarkers of depression from an individual perspective using the proposed method.

Sex is usually a factor of interest in the study of brain science, therefore our work provides some exploration of the specificity of sex in age prediction. The results show that the specificity of sex in age prediction is not significant. The primary reason is that sex has few effects on GMV. Indeed, differences in brain anatomy between males and females have received extensive attention, but the findings are inconsistent or even contradictory. For example, there are inconsistent findings on the gray and white matter structures [55,56,57] (hippocampus: F > M [55], F ≈ M [56], F < M [57]). Recent findings suggest that many factors contribute to the inconsistency, with differences in body and head size between males and females being a major factor; when the effects of these factors are removed, there is little difference in GMV between males and females [33]. In our study, all subjects had been registered to the standard MNI152 template, thus eliminating anatomical size differences.

It should be noted that our study has two limitations: one is that the prediction accuracy needs to be improved, and the other is that only MRI data is used, which limits its application. Although the prediction accuracy of the proposed algorithm is better than the classical algorithm, it still has a certain gap compared with the deep learning method. Improving the prediction accuracy can be attempted in two aspects in the future: one is to start from the feature engineering perspective to obtain more representative features; the other is to try to use new models such as deep learning and explore the interpretability of the models. In addition, a multimodal data format can be planned in order to make our method have a wider range of use. For example, the diagnosis of AD and the search for biomarkers can be investigated from different perspectives such as MRI, fMRI, and DTI using the OASIS-3 dataset [58], which will help to discover the functional and structural correspondence of the patient’s brain. In summary, notwithstanding its limitation, this study does suggest its potential and possibility address a wide range of problems. The relationship between targets and brain structures is explained while achieving target prediction.

## 5. Conclusions

Based on the interpretable SHAP model, the relationship between individual age and gray matter volume is investigated in this paper. First, in the case of all subjects, the age prediction accuracy based on XGBoost was 7.77 years, and then the relationship between age and gray matter was investigated. This work studied the relationship between age and gray matter separately regarding both gray matter itself and their interaction network. Globally, it was first found that only specific gray matter locations had a high correlation with age. For individuals, different locations of gray matter were found to play different roles in age, with some gray matter pushing up age and others pulling down age. Moreover, the role of these gray matter changes with age. In addition to the gray matter itself, it also found that the interaction between gray matter was closely related to age. Furthermore, by studying the dynamic properties of individuals as they age, it is revealed that gray matter and its interactions are dynamically associated with age.

The proposed method is a data-driven strategy and thus can be used to address similar problems. In future work, we intend to use the approach to address the classification of degenerative brain diseases, such as Alzheimer’s and Parkinson’s diseases. On the basis of achieving classification, we will try to find global and individual biomarkers to support the treatment and diagnosis of these diseases.

## Figures and Tables

**Table 1:** Age prediction results reported in the literature.

Articles	Method	MAE (Years)
[12]	CNN * (FPN *)	5.55
	GPR * (FPN)	7.47
	RVR * (FPN)	7.76
[14]	CNN (GM *)	4.16
	GPR (GM)	4.66

* CNN, Convolutional Neural Networks; FPN, Frontoparietal Network; GPR, Gaussian Process Regression; RVR, Relevance Vector Regression; GM, Gray Matter.

## Data Availability

Data is available from the website https://www.oasis-brains.org/ accessed on 9 November 2022.
